# Ten-Year Results of Inguinal Hernia Open Mesh Repair

**DOI:** 10.3389/jaws.2025.14384

**Published:** 2025-06-09

**Authors:** Ceith Nikkolo, Toomas Lillsaar, Tiit Vaasna, Ülle Kirsimägi, Urmas Lepner

**Affiliations:** ^1^ Surgery Clinic, Tartu University Hospital, Tartu, Estonia; ^2^ Faculty of Medicine, University of Tartu, Tartu, Estonia; ^3^ Radiology Clinic, Tartu University Hospital, Tartu, Estonia

**Keywords:** inguinal hernia, Lichtenstein repair, self-gripping mesh, hernia recurrence, chronic pain

## Abstract

**Introduction:**

The primary aim of the present prospective study was to determine the clinical recurrence rate of inguinal hernia at a 10-year follow-up. Other aims included evaluating the recurrence rate based on ultrasound (US) examination and assessing chronic pain and foreign body feeling following open inguinal hernia repair.

**Methods:**

A pain questionnaire was completed 10 years after surgery. At the follow-up visit, the patients were examined for a recurrent hernia. For patients who completed a 10-year follow-up and did not have clinical hernia recurrence, an ultrasound of the inguinal canal was performed in addition to a clinical examination.

**Results:**

The data of 242 patients were analyzed at 10-year follow-up. At the 3-year follow-up, three clinical recurrences were diagnosed. Additionally, two recurrences were diagnosed between the 6-month and 3-year follow-up visits, and three were diagnosed between the 3-year and 10-year follow-up visits. At the 10-year follow-up visit, seven recurrences were clinically diagnosed, and in twenty-three cases, ultrasound detected recurrent inguinal hernias that were not clinically detectable. Of the patients, 94.5% (95% CI 91.8%–97.2%) are recurrence-free at the 10th postoperative year. Of the patients, 18.6% (95% CI, 14.0–24.2) experienced pain in the inguinal area during various activities, including at rest, upon coughing, when rising from a lying to a sitting position, and during physical activities. The mean VAS score was 37.6 (SD 21.5), based on the highest VAS score during different activities. Of the fifteen patients with clinically recurrent hernias, 66.7% reported pain during various activities. In contrast, the rate of chronic pain among patients without clinical hernia recurrence was significantly lower at 15.4% (p < 0.001). Of the twenty-three patients with US recurrence, 17.4% experienced pain in the inguinal area. The respective result among patients without US and clinical recurrence was 15.2% (p = 0.763). Foreign body feeling was reported by 26 patients (12.8%) without hernia recurrence, by three patients (20%) with clinical recurrence, and by two patients (8.7%) with ultrasound recurrence (p = 0.560).

**Conclusion:**

Considering the high rate of late recurrences, a follow-up of at least 10 years is necessary to determine the accurate recurrence rate after open inguinal hernia mesh repair. Further studies are needed to clarify the significance of US recurrences.

**Clinical Trial Registration:**

clinicaltrials.gov, identifier NCT06008535.

## Introduction

Although the focus of inguinal hernia surgery has shifted towards laparoscopic repairs, Lichtenstein hernioplasty is likely to remain a viable option. According to the updated international inguinal hernia guideline, for patients with primary unilateral inguinal hernia, a laparoscopic technique is recommended due to its lower incidence of postoperative pain and a reduction in chronic pain. However, specific patient and hernia characteristics, such as those following prostatic surgery, pelvic radiation, or scrotal hernia, may warrant Lichtenstein repair as the first choice [1].

Many studies over the last two decades have focused on the rate of chronic pain after inguinal hernia surgery and the search for an ideal mesh, while the recurrence rate has been somewhat neglected. The possible cause for the recurrence rate being often a secondary research question is its reported low rate after inguinal hernia repair. A 2018 meta-analysis reported recurrence rates of 2.1% after the Lichtenstein operation, 1.8% after totally extraperitoneal (TEP) repair, and 1.9% after transabdominal pre-peritoneal (TAPP) repair [2]. However, some database and registry-based studies have demonstrated that up to 11% of inguinal hernia repairs are performed for recurrences [3, 4]. Based on those results, Murphy et al. concluded that the current literature on inguinal hernia recurrences is overly optimistic [3].

The primary aim of the present study was to determine the clinical recurrence rate of inguinal hernia at a 10-year follow-up. Other aims included evaluating the recurrence rate based on ultrasound (US) examination and assessing chronic pain and foreign body feeling following open inguinal hernia repair.

## Methods

The present study is a 10-year prospective follow-up study involving patients from three randomized studies, the detailed methods (including randomization processes) of which have been published previously [5–7].

This study was reported in accordance with the STROBE checklist to ensure transparency and completeness in reporting [8].

### Inclusion and Exclusion Criteria

Eligible patients who consented to participate in the study were adults aged 18 years or older undergoing elective unilateral hernia surgery for primary reducible inguinal hernia. Patients younger than 18 years and those with irreducible, strangulated, or recurrent hernias were excluded. Additionally, patients who were unable to understand the questionnaire or were unwilling to participate in the study were excluded from the analysis.

The patients’ enrollment periods were between January 2007 and July 2008, January 2011 and April 2012, and January 2012 and June 2013.

### Meshes and Operation Techniques

Four different meshes were used: Premilene^®^ Mesh (B. Braun), Optilene^®^ Mesh LP (B. Braun), Ultrapro™ Mesh (Ethicon), and Parietex ProGrip™ Mesh. Premilene^®^ Mesh is a monofilament polypropylene mesh weighing 82 g/m^2^ with a pore size of 0.8 mm. Optilene Mesh LP is a monofilament polypropylene mesh, weighing 36 g/m^2^, with a pore size of 1.0 mm. Ultrapro™ Mesh is a lightweight, partially absorbable mesh composed of polypropylene and poliglecaprone, with a weight of 28 g/m^2^ and a pore size of 3–4 mm. Parietex ProGrip™ Mesh is a partially absorbable monofilament mesh consisting of polyester and polylactic acid weighing 38 g/m^2^ after resorption and with a pore size of 1.1 × 1.7 mm. Parietex ProGrip™ Mesh has microgrips across the mesh area, which ensure gripping between muscle fibers and the connective tissue. All meshes, except Ultrapro™ Mesh, were commercially preshaped. Ultrapro™ Mesh was shaped by the surgeon during the operation using a stencil, and a mesh with measurements 4.5 × 10 cm was applied. Premilene^®^ Meshes used in this study were with measurements 4.5 × 10 cm, Optilene^®^ Mesh LP meshes were with sizes 4.5 × 10 cm or 6 × 14 cm, and Parietex ProGrip™ Meshes were with measurements 8 × 12 cm.

In the cases of Premilene^®^ Mesh, Optilene^®^ Mesh LP, and Ultrapro™ Mesh, a Lichtenstein repair was performed. The polypropylene 2/0 suture material was used for mesh implantation. For self-gripping mesh, the inguinal canal was prepared, and the wound was subsequently closed, as in a Lichtenstein repair. The mesh was placed in position in the inguinal canal, the flaps were closed around the cord, and pressure was applied to the mesh to secure it in place.

The patients were blinded to the type of mesh they received.

### Documented Data

The preoperative and postoperative data were documented using standardized forms. The patient study form included demographic data, body mass index, type of hernia (medial or lateral), size of the hernia, handling of the hernial sac, type of mesh used, duration of the operation, and length of hospital stay.

### Follow-Up Visits

Follow-up visits took place 7 days, 1 month, 6 months, 3 years, and 10 years after surgery.

A pain questionnaire was completed at every follow-up visit. The questionnaire included questions about pain at rest, coughing, rising from a lying to a sitting position, and during physical effort and exercise (all yes-or-no questions). When the patient’s response to the questionnaire was positive, the pain scores were measured on a visual analog scale (VAS) ranging from 0 (no pain) to 100 mm (worst imaginable pain). The patients were also asked whether the pain influenced their everyday activities and whether they used analgesics for inguinal pain.

Foreign body feeling was registered as a yes-or-no question.

Additionally, it was specified whether the patient found the sensation in the operation area normal.

At short-term follow-up visits, patients were examined for any signs of wound complications, and a clinical examination was conducted at every visit to assess for the recurrence of hernia.

### Primary Endpoint

The primary endpoint of this study was the recurrence rate, considering all patients who had developed recurrence by the 10th year. The patients were examined for a recurrent hernia at every follow-up visit. For patients who completed a 10-year follow-up and did not have clinical hernia recurrence, an ultrasound of the inguinal canal was performed in addition to a clinical examination. The ultrasound examination was conducted by the same certified radiologist using a GE Logiq E9 ultrasound machine (GE Healthcare) with a 9L-D linear transducer. Examination of the inguinal region was initially performed with the patient in a supine position. The Valsalva maneuver was also used to identify transient hernias. If no hernia was detected, the patient was also studied while standing. The inguinal area was scanned in two different planes. The criterion for recurrence of inguinal hernia was the detection of herniated abdominal tissue inside the inguinal canal or protrusion of abdominal contents within Hesselbach’s triangle. The ultrasound technique used is described in more detail in a previously published article by Jamadar et al. [9].

Patients who developed a recurrent hernia between follow-up visits were included in the analysis using the “carrying forward” method, which utilized the data from the last available follow-up visit.

### Statistical Analysis

The software package Statistica, version 13.3 (TIBCO Software Inc., USA), was used for statistical analysis. Continuous data are presented as means with standard deviations (SD) for normally distributed variables, while non-normally distributed variables are presented as medians along with the 25th and 75th percentiles. Categorical variables were presented in counts and percentages. Comparison between groups was made using Pearson’s Chi-square or Fisher’s exact test for categorical data. The Kaplan-Meier method was utilized to estimate recurrence-free survival, allowing for the assessment of the time interval during which patients did not experience hernia recurrence. The log-rank test was used to compare recurrence-free survival in different mesh groups. All statistical tests were two-sided; p < 0.05 was considered statistically significant.

## Results

The patients included in the study were operated on at the Surgery Clinic of Tartu University Hospital between January 2007 and July 2008, January 2011 and April 2012, and January 2012 and June 2013. Four hundred twenty-three patients were enrolled in the initial study groups. The preoperative data, the intraoperative data, and the short- and midterm results have been previously published [5–7, 10–12].

**FIGURE 1:**
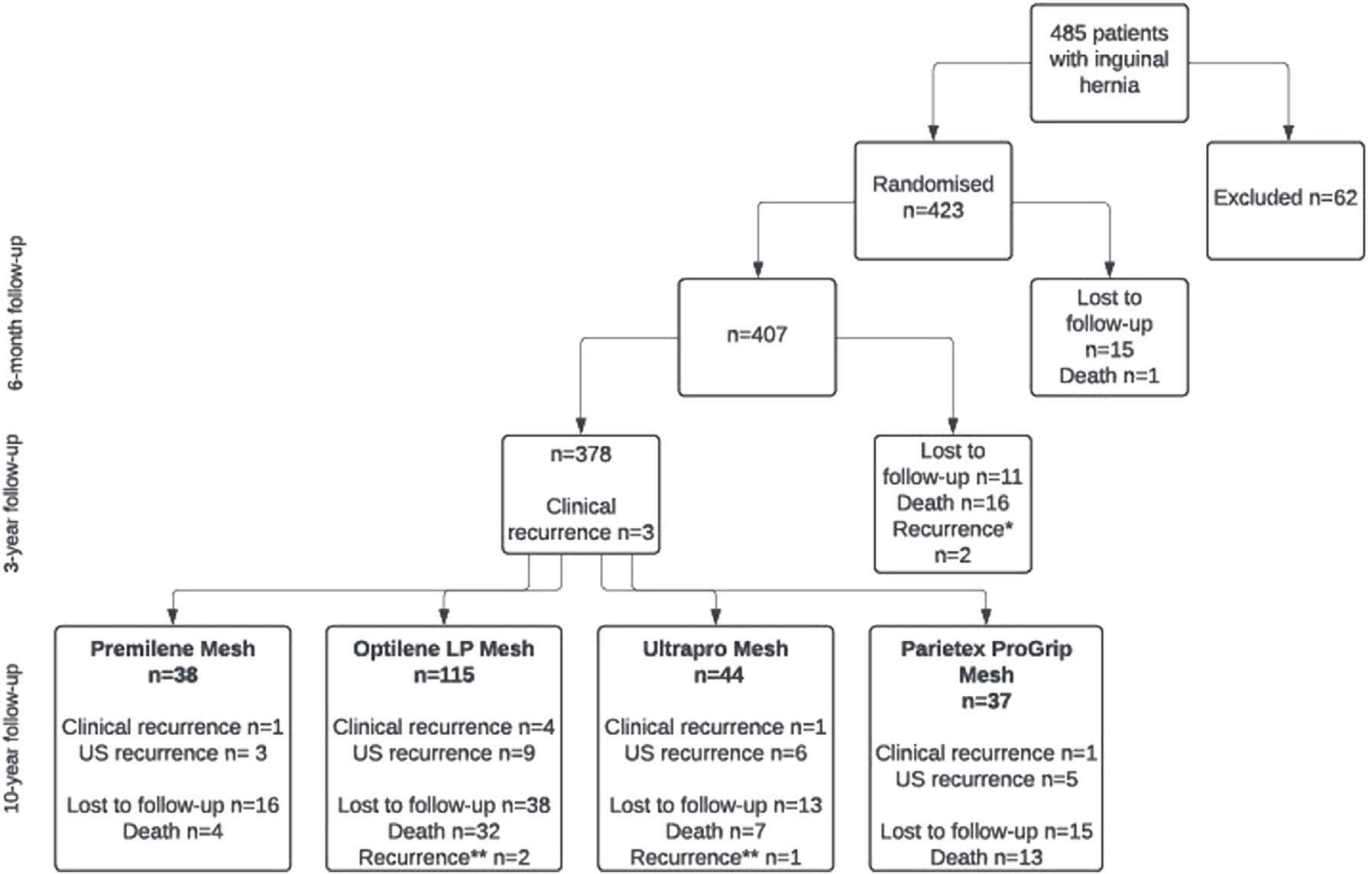
Study flowchart. *Recurrence diagnosed between 6-month and 3-year follow-up visits. **Recurrence diagnosed between 3-year and 10-year follow-up visits.

The 10-year follow-up visits took place from April 2018 to December 2023. The data of 242 patients were analyzed at 10-year follow-up (234 patients who completed the 10-year follow-up + 8 patients with “carrying forward” data) (Figure 1). One hundred eight patients were lost, and seventy-three patients died during the follow-up period (drop-out rate 42.8%). The causes of death were non-hernia surgery-related. Initial operative data of patients included in the 10-year analysis are presented in Table 1.

**TABLE 1:** Initial patients’ and operation data.

Patients’ and operation data	n = 242
Mean age, years (SD)	54.9 (14.6)
Sex, n (%) Female Male	18 (7.4) 224 (92.6)
Mean BMI, kg/m^2^ (SD)	25.8 (3.3)
Hernia, n (%) Medial Lateral Combined	86 (35.5) 141 (58.3) 15 (6.2)
Size of the defect (cm), n (%) <1.5 1.5–3 >3	56 (23.1) 148 (61.2) 38 (15.7)
Anesthesia, n (%) Spinal Laryngeal mask Endotracheal	30 (12.4) 196 (81.0) 16 (6.6)
Mean operating time, min (SD)	48.7 (14.0)
Hernial sac handling, n (%) Resected Nonresected	151 (62.4) 91 (37.6)

BMI, body mass index; SD, standard deviation.

**FIGURE 2:**
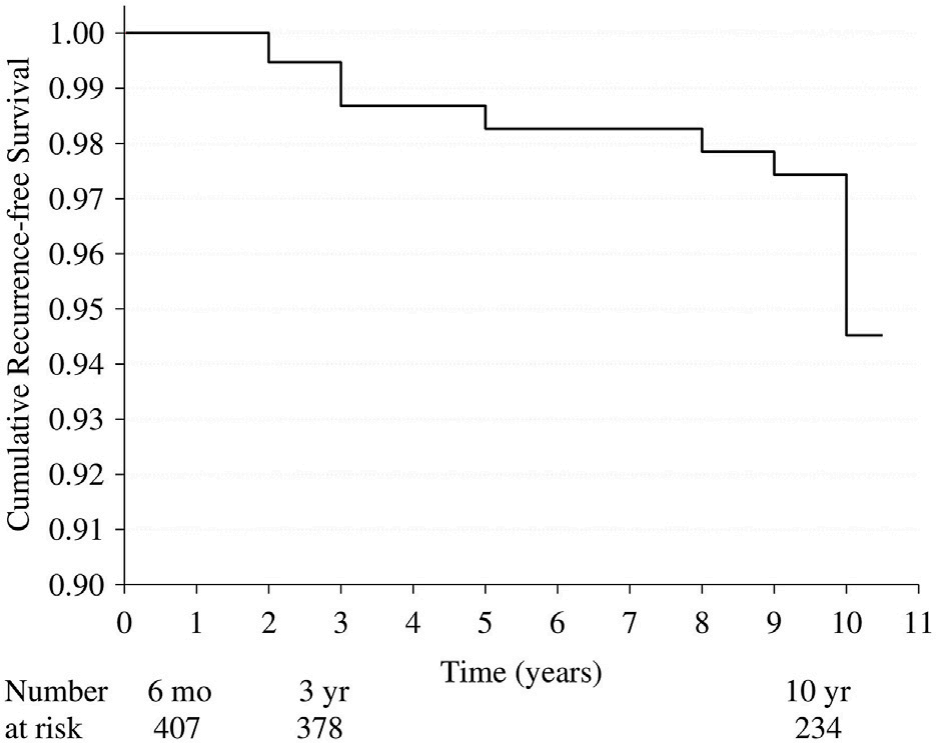
Kaplan-Meier cumulative recurrence-free survival rate based on clinical recurrences. 6 mo 6-month follow-up, 3yr 3-year follow-up, 10 yr 10-year follow-up.

No hernia recurrences were found at the 6-month follow-up. At the 3-year follow-up, three clinical recurrences were diagnosed. Additionally, two recurrences were diagnosed between the 6-month and 3-year follow-up visits, and three were diagnosed between the 3-year and 10-year follow-up visits. At the 10-year follow-up visit, seven recurrences were clinically diagnosed, and in twenty-three cases, ultrasound detected recurrent inguinal hernia that was not clinically detectable. Figure 2 presents cumulative recurrence-free survival based on clinical recurrences. Of the patients, 94.5% (95% CI 91.8%–97.2%) are recurrence-free at the 10th postoperative year.

Table 2 illustrates the recurrence rates based on sex, hernia type (medial, lateral, or combined), hernia size, and various meshes. In the clinical recurrence group, more patients initially had lateral hernia (60% and 40%, respectively). In the US recurrence group, there were also more patients with lateral hernias (26.1% medial hernias, 70% lateral hernias, 4.3% combined hernias). According to the Kaplan-Meier analysis, there were no significant differences in recurrence rates among the different mesh groups (p = 0.474).

**TABLE 2:** Recurrences based on sex, hernia characteristics, and different meshes.

Patients’ and operation data	No recurrence	Clinical recurrence	Ultrasound recurrence	p-value^a^
Sex, n (%) Female Male	15 (83.3) 189 (84.4)	1 (5.6) 14 (6.3)	2 (11.1) 21 (9.4)	0.878
Hernia, n (%) Medial Lateral Combined	74 (86.1) 116 (82.3) 14 (93.3)	6 (7.0) 9 (6.4) 0 (0)	6 (7.0) 16 (11.4) 1 (6.7)	0.802
Size of the defect (cm), n (%) <1.5 1.5–3 >3	46 (82.1) 128 (86.5) 30 (79.0)	4 (7.1) 6 (4.1) 5 (13.2)	6 (10.7) 14 (9.5) 3 (7.9)	0.330
Meshes, n (%) Premilene^®^ Mesh Optilene^®^ Mesh LP Ultrapro™ Mesh Parietex ProGrip™ Mesh	34 (87.2) 102 (84.3) 37 (87.2) 31 (83.8)	2 (5.1) 10 (8.3) 2 (4.4) 1 (2.7)	3 (7.7) 9 (7.4) 6 (13.3) 5 (13.5)	0.737

^a^ Fisher’s exact test.

Table 3 presents positive responses to the pain questionnaire. Among the patients, 18.6% (95% CI, 14.0–24.2) experienced pain in the inguinal area during various activities, including at rest, upon coughing, when rising from a lying to a sitting position, and during physical activities. The mean VAS score was 37.6 (SD 21.5), based on the highest VAS score during different activities.

**TABLE 3:** Positive answers to the pain questionnaire at 10-year follow-up.

Pain in the groin during different activities	No recurrence n = 204	Clinical recurrence n = 15	Ultrasound recurrence n = 23	p-value^a^
At rest	0 (0%)	1 (6.7%)	0 (0%)	0.062
At coughing	2 (1%)	2 (13.3%)	0 (0%)	0.029
When rising from lying to sitting	7 (3.4%)	3 (20%)	1 (4.4%)	0.033
During physical activity	29 (14.2%)	10 (6.7%)	4 (17.4%)	<0.001

^a^ Fisher’s exact test.

Of the fifteen patients with clinically recurrent hernias, 66.7% reported experiencing pain during various activities. In contrast, the rate of chronic pain among patients without clinical hernia recurrence was significantly lower at 15.4% (p < 0.001). Of the twenty-three patients with US recurrence, 17.4% experienced pain in the inguinal area. The respective result among patients without US and clinical recurrence was 15.2% (p = 0.763).

In Table 4, the presence of pain at rest or during various activities is presented across different periods. Among patients who experienced pain at every follow-up visit, the clinical recurrence rate was higher compared to patients who reported no pain at follow-up visits (17.4% vs. 3.1%, respectively; p = 0.024).

**TABLE 4:** Positive answers to the pain questionnaire through different periods.

Patients, n	6-month follow-up	3-year follow-up	10-year follow-up	Recurrences, n	
				Clinical	US
98	-	-	-	3	6
56	+	-	-	2	6
28	+	+	-	0	4
15	-	+	-	0	3
5	+	-	+	1	0
9	-	+	+	2	0
8	-	-	+	3	0
23	+	+	+	4	4

“+” positive answer (i.e., pain present) to pain questionnaire at rest or during any activities, “-” negative answer (i.e., no pain) to pain questionnaire at rest or during any activities, *US* ultrasound.

Four patients (2%) without clinical hernia recurrence used analgesics for inguinal pain. The respective results among patients with clinical and ultrasound hernia recurrence were 1 (6.7%) and 2 (8.7%), respectively (p = 0.080). Groin pain influenced patients’ everyday activities in 5 cases (2.5%) without clinical hernia recurrence, in 3 cases (20%) with clinical hernia recurrence, and in 2 cases (8.7%) with ultrasound hernia recurrence (p = 0.005).

Foreign body feeling was reported by 26 patients (12.8%) without hernia recurrence, by three patients (20%) with clinical recurrence, and by two patients (8.7%) with ultrasound recurrence (p = 0.560).

Of the patients without hernia recurrence, 94.1% reported normal feeling in the groin area. The results for patients with clinical recurrence and ultrasound recurrence were 73.3% and 91.3%, respectively (p = 0.021).

## Discussion

The primary outcome of our study was the recurrence rate of inguinal hernia open mesh repair at 10-year follow-up, which, based on clinical examination, was 6.2%. According to a recently published high-quality systematic review and updated network meta-analysis, the recurrence rate for Lichtenstein repair is 2.1% [2]. However, there are several studies in which the proportion of operations performed due to inguinal hernia recurrences in male patients is 9%–11% [3, 13], which suggests a much higher recurrence rate. Based on these findings of recurrent hernia surgery, Murphy et al. even concluded that the current literature on inguinal hernia recurrence is overly optimistic [3]. A systematic review by Aiolfi et al. included studies with shorter follow-up periods (ranging from 13 months to 7 years) [2], compared to our study. Considering that 46.7% of clinical recurrences were diagnosed at 10-year follow-up, more extended follow-up periods are probably needed to find true recurrence rates after hernia repair. Köckerling et al. found in their registry-based study that even a ten-year follow-up is insufficient, as only 57.5% of inguinal hernia recurrences occurred within this timeframe. They concluded that to determine the actual recurrence rate of inguinal hernia repair, a fifty-year follow-up for inguinal hernia surgery would be necessary [14], which the present study’s authors consider to be exaggerated.

Early hernia recurrences are typically associated with technical failures [13], whereas late recurrences are more often influenced by patient-related factors [14, 15]. Although we experienced many late recurrences, unfortunately, we did not collect data on patient-related factors (e.g., changes in body mass over 10 years, smoking habits, new comorbidities, etc.).

At follow-up visits, we diagnosed 15 hernia recurrences during clinical examination. However, at a 10-year follow-up, a US examination of the groin area was performed on patients who did not have hernia recurrence in clinical examination, revealing 23 recurrent hernias that were not clinically detectable. Although the US recurrence rate is worrisome, considering that 91.3% of patients with US recurrence reported normal feeling in the groin area, and chronic pain was reported among US recurrence patients as often as among patients without US and clinical recurrence, US recurrence cases are likely clinically insignificant. The rate of incarceration/strangulation of inguinal hernias is estimated to be 0.3%–3% per year [16]; however, little is known about the natural evolution of asymptomatic occult hernias [1]. So, we can only speculate whether, over the following years, those recurrences would become a clinical problem.

According to Köckerling et al., the incidence of spermatic cord lipoma ranges from 20% to 70% of all inguinal hernia repairs. If it is missed during operation, it can be diagnosed as a recurrence or pseudo-recurrence postoperatively on ultrasound [17]. Unfortunately, in our study, we did not record whether a cord lipoma was present or not. However, we can speculate that some recurrences diagnosed on US could be unresected cord lipomas rather than true recurrent hernias.

When considering the cause of recurrences, mesh shrinkage has to be taken into account. Shrinkage is the process by which the mesh contracts due to the formation of scar tissue [18]. Although most of the meshes used in our study were polypropylene meshes, which have less shrinkage compared to other mesh materials [19], the meshes we used were very small in measurement, which, after the shrinkage process, can increase the likelihood of hernia recurrence development.

Gutlic et al. demonstrated similar rates of chronic pain at 3-year and 8-year follow-up visits [19]. Although the rate of chronic pain at a 10-year follow-up in our study is higher than in the Gutlic research, compared to our mid-term results, the rate of chronic pain has decreased over time [10–12]. Still, 18.6% of the patients reporting pain 10 years after inguinal hernia surgery is thought-provoking. As expected, the rate of pain was higher among patients with clinical hernia recurrence. Considering that, in patients without clinical hernia recurrence, the pain did not significantly influence their everyday activities, and analgesic consumption was low, we can hypothesize that quality of life is not significantly affected by groin pain.

In the Paajanen et al. study, the rate of foreign body feeling at a 10-year follow-up after the Lichtenstein operation was 11.3% [20]. Similarly, our research also reported a comparable rate of foreign body feeling among patients without recurrent hernia (12.8%). Among patients with clinical recurrence, the rate of foreign body feeling was higher, but the difference was not statistically significant.

A limitation of the present study is that it is a single-center study, which may bias the long-term results. Additionally, the high drop-out rate may also bias the results. Considering the well-developed medical databases and the relatively small distances in our country, it was surprising to the authors that so many patients were lost due to difficulties in reaching them or their unwillingness to participate, often because of changes in their residence.

In conclusion, considering the high rate of late recurrences, a follow-up of at least 10 years is necessary to determine the accurate recurrence rate after open inguinal hernia mesh repair. Further studies are needed to clarify the significance of ultrasound recurrences.

## Data Availability

The raw data supporting the conclusions of this article will be made available by the authors, without undue reservation.
